# 

**Published:** 1857-06

**Authors:** 


### CONTENTS.

ORIGINAL COMMUNICATIONS— PAGE.

An Address delivered at the First Annual Meeting of the DeWitt County Medical Society, held at Clinton, Ill. April 6, 1857. By Christopher Goodbrake, M.D. Published by request of the Society.................. 247

Iodine in Scrofula, Phthisis, Pulmonalis, and all that class of Diseases, which depend on an hereditary tendency or constitutional taint. By B. Woodward, M.D., Galesburg, Ill.......................................... 257

The Pathology of Milk-Sickness, in Parke County, Ind. By Dr. John Pickard, ................................................................ 260

Opthalmia, Ulceration of the Cornea, being the Substance of a Clinical Lecture to the Class attending the Mercy Hospital; delivered in Jan. 1857. By N. S. Davis, M.D. Prof. of Prin. and Practice of Med. and Clin. Med. ................................................................ 262

SELECTIONS—

Dr. Edward Brown-Sequard's Experimental and Clinical Researches, Epilepsy, (Continued)................................................ 272

BOOK NOTICES—

Clinical Lectures on Certain Diseases of the Urinary Organs; and on Dropsies. By Robert Bently Todd, M.D., F.R.S......................... 284

Transactions of the American Medical Association, Vol. IX. 1856........ 290

EDITORIAL—

American Medical Association................................................ 191

Cook County Medical Society ................................................ 292

To CORRESPONDENTS .......................................................... 292

